# Label-Free Electrochemical Detection of K-562 Leukemia Cells Using TiO_2_-Modified Graphite Nanostructured Electrode

**DOI:** 10.3390/bios16010028

**Published:** 2026-01-01

**Authors:** Martha Esperanza Sevilla, Rubén Jesús Camargo Amado, Pablo Raúl Valle

**Affiliations:** 1 Faculty of Systems, Electronics and Industrial Engineering, Technical University of Ambato, Ambato 180101, Ecuador; 2 School of Chemical Engineering, Universidad del Valle, Cali 76001, Colombia; ruben.camargo@correounivalle.edu.co; 3 Faculty of Civil and Mechanical Engineering, Technical University of Ambato, Ambato 180101, Ecuador; prvalle@uta.edu.ec

**Keywords:** TiO_2_-m electrodes, pyrolytic graphite, chronic myeloid leukemia, cancer

## Abstract

This manuscript presents the development of an electrochemical biosensor designed to detect K-562 chronic myeloid leukemia (CML) cells. The biosensor was made of highly oriented pyrolytic graphite (HOPG), functionalized with -OH and -COOH groups by surface etching with strong acids, and subsequently coated with modified titanium dioxide (TiO_2_-m). TiO_2_-m is TiO_2_ modified during its synthesis process using carbon nanotubes functionalized with -OH and -COOH groups. These changes improve the electron transfer kinetics and physicochemical properties of the electrode surface. TiO_2_-m improves the sensitivity and selectivity towards leukemic cells. The detection process involved three stages: cell culture, cell adhesion onto the TiO_2_–m electrode, and measurement of the electrochemical signal. Fluorescence microscopy and SEM-EDS confirmed cell adhesion and pseudopod formation on the TiO_2_-m surface, which is an important finding because K-562 cells are typically nonadherent. Cyclic voltammetry (VC) and differential pulse voltammetry (VDP) demonstrated rapid and sensitive detection of leukemic cells within the concentration range of 6250 to 1,000,000 cells/mL, achieving high reproducibility and strong linearity (R^2^ = 98%) with a detection time of 25 s. The VC and VDP demonstrated rapid and sensitive detection of leukemic cells over a concentration range of 6250 to 1,000,000 cells/mL, achieving adequate reproducibility and stable linearity (R^2^ = 98%), with a detection time of 25 s. These results indicate that the TiO_2_-m biosensor is a promising platform for the rapid and efficient electrochemical detection of leukemia cells.

## 1. Introduction

Cancer remains the second leading cause of mortality worldwide and is associated with a low median survival rate [1], underscoring the need for rapid, cost-effective, and accessible diagnostic technologies. Recent advances in medical technology have accelerated the development of biosensors capable of providing early cancer detection with high precision and simplicity in use. CLM is a myeloproliferative neoplasm characterized by a specific genetic abnormality [2] and represents a critical target for such innovations [3,4]. CML originates in the bone marrow and leads to the overproduction of abnormal white blood cells, driven by chromosomal alterations involving the Philadelphia chromosome [5] which carries the pathogenic BCR–ABL fusion gene [6,7].

Electrochemical biosensors can transform the interaction of biomolecules or biological signals into electrical signals [7,8,9,10] offering high sensitivity and compatibility with nanostructured detection interfaces for both in vitro and in vivo applications [7,8,9,10,11].

The nanoparticles have sizes ranging from 1 to 100 nm [8,12]. In addition, the semiconductor nanomaterials successfully used in biosensors are: TiO_2_ [13], TiO_2_/GO [10,14,15] gold nanoparticles [13,16], nanoparticles (ZnO NPs) [17], CNT nanoelectrodes [18] CdS quantum dots (QD), titanium dioxide sol/graphene nanocomposite [19], nano QD CdSe, QD CdTe, N-doped graphene QD [19,20].

TiO_2_ is a wide-bandgap [21] semiconductor (3.2 eV) [22] known for its exceptional photocatalytic activity [23], biocompatibility, chemical stability, and low toxicity [24]. TiO_2_-m demonstrated selective cytotoxicity toward cancer cells, making it promising for diagnostic and therapeutic applications (patent WO2016055869A1) [25,26].

This study presents the development and validation of a K-562 cell electrochemical biosensor, including surface characterization by FTIR, SEM–EDS, fluorescence microscopy [27,28] and electrochemical evaluation via cyclic and differential pulse voltammetry.

## 2. Materials and Methods

### 2.1. Chemicals

Sulfuric acid 97% ACS Reagent (Honeywell Fluka, Charlotte, NC, USA), Nitric Acid 65%, (Merck, Darmstadt, Germany), hydrochloric acid 37.5%, Ammonium Hydroxide Certified A, CS, Plus A669C-212/Fisher, and HOPG, (Carbon Industry Co., Ltd., Taiyuan, China) with porosity 0.8 max, density 1.85 g/cm^3^, compressive strength 65 MPa, and flexural strength 45 MPa were used. Modified titanium dioxide TiO_2_-m; Dulbecco Modified Iscove Medium [+] (GIBCO, Grand Island, NY, USA); RPMI 1640 with L-glutamine (GIBCO, Grand Island, NY, USA), HEPES buffer (25 mM) (SIGMA^R^, Burlington, MA, USA); sodium bicarbonate (GIBCO, Grand Island, NY, USA); sodium bicarbonate (SIGMA^R^, Burlington, MA, USA); gentamicin; 10% fetal bovine serum (GIBCO, Grand Island, NY, USA); and CML K-562 cells (ATCC CCL-243, Manassas, VA, USA).

### 2.2. Preparation of the GO/HOPG Electrode

HOPG plates with a thickness of 1.5 mm, density of 1.85 g/cm^3^, and compressive strength of 65 MPa were used. HOPG was cleaned with deionized distilled water and then, ultrasonicated for 15 min in 70% ethyl alcohol and coated by spraying a mixture of two strong acids (sulfuric acid and nitric acid) in a 3:1 ratio. After 10 min, 2/3 of HCl was added at 2, 4, and 6 h. Throughout the process, neutralization was carried out with 5% NH_4_OH, resulting in plates functionalized with GO/HOPG on their surfaces [27].

The acid spray-coating method is known to induce surface oxidation on HOPG, generating various oxygen-containing functional groups (e.g., hydroxyl and carboxyl groups) and creating a roughened surface topography. The oxidized graphite layer (GO/HOPG) provides enhanced sites for the subsequent adhesion of the TiO_2_-m layer. The term “GO/HOPG” reflects the presence of graphite oxide-like functional groups rather than a separate graphene oxide coating.

### 2.3. Preparation of Modified TiO_2_

The modified TiO_2_-m was synthesized using the sol–gel method, with titanium (IV) n-butoxide as a precursor. This was dissolved in anhydrous ethanol, and hydration upon contact with the environment was avoided. The solution was kept under constant magnetic stirring for 15 min, and then deionized water was added dropwise until gelation occurred. (detailed description of the synthesis method for TiO_2_-m is published in patent number US20170281770A1 [29]).

### 2.4. Preparation of Electrode TiO_2_-m/GO/HOPG

A TiO_2_-m layer was deposited onto the GO/HOPG electrode via impregnation. After coating, the electrode underwent heat treatment using a calcination ramp of 8.46 °C/min from room temperature to 150 °C and a holding time of 30 min to reduce the internal stresses generated by solvent accumulation. The temperature was then increased to 11.66 °C/min to 500 °C and held for 45 min. The electrodes were then allowed to rest, resulting in TiO_2_-m/GO/HOPG plates being formed.

To simplify the nomenclature, an alphanumeric code (TGO360) was used, where the first letter “T” represents TiO_2_-m, and the following letters represent GO/HOPG. The first digit represents the acid used (sulfuric, nitric, or hydrochloric), the second digit represents the acid contact time in hours, and the third digit represents the resting time in days of the TiO_2_-m sol–gel. TGO360 is HOPG treated with three acids for 6 h and surface-impregnated with TiO_2_-m sol–gel without any resting time. In the present study, an electrochemical biosensor was developed using a HOPG electrode [25,26] functionalized using an acid spray-coating method [27]. This process introduces functional groups -OH and -COOH onto the surface of the HOPG [28,29,30], which improve the performance of the TGO360 electrode K-562 cells, which are normally non-adherent, adhered strongly to the modified surface, as confirmed by fluorescence microscopy and SEM imaging. This adhesion contributed to the improved electrochemical response of the biosensor, resulting in a signal.

### 2.5. Cell Culture Medium

K-562 (ATCC CCL-243) CML cells were cultured in Iscover Modified Dulbecco’s medium (Gibco) supplemented with 5% fetal calf serum and 1% gentamicin at 37 °C in a humidified atmosphere. with 5% CO_2_. The cells were cultivated in Iscover medium with fetal bovine serum and 1% gentamicin. The trypan blue test was used to determine the cell counts [23].

### 2.6. Cell Culture

K-562 CML cells were cultured in a flask containing Iscover ATCC medium, 10% fetal calf serum, and 50ug/mL gentamicin at 37 °C and 5% CO_2_. Cell counting was performed using a Neubauer chamber. In an Eppendorf, 100 µL of K-562 cells were mixed with 400 µL of trypan blue at a ratio (1:4). From this mixture, 10 µL of cell suspension was taken, placed in the chamber, and the cell count was determined.

Once the cell count was performed and the number of cells contained in the box was determined, the cells were centrifuged in a falcon tube at 1250 rpm for 10 min. Different dilutions were prepared at different concentrations to perform electrochemical biosensor testing. The concentrations used in the biosensor tests vary from 1 × 10^6^ cells/mL, 5 × 10^5^ cells/mL, 1.0 × 10^5^ cells/mL, 2.5 × 10^4^ cells/mL, 6250 cells/mL, 1562 cells/mL.

### 2.7. Cell Fixation on TGO360 Plates

A concentration of 1 × 106 cells/mL was placed on the TGO360 and pyrolytic graphite plates and incubated for 24 h at 37 °C and 5% CO_2_ in an air atmosphere. After 24 h, the supernatant was removed with a 5mL pipette, washed with 0.1M phosphate-buffered saline PBS–2–3 times to remove cells and cell debris that did not adhere to the surface of the plates. Cell fixation with glutaraldehyde was performed for 1 h, and the glutaraldehyde-coated plates were placed at 4 °C in the refrigerator. The plates were then washed with PBS 0.1 M (pH 7.4) for 10 min each, and the cells were dehydrated with 50%, 70%, 80%, 95%, and 100% alcohol each for 10 min intervals.

### 2.8. DAPI Staining

The cells found on the surface of the TGO360 plates were stained with 4-6-Diamidin-2-Phenylindole (DAPI), 1 mL was added with a syringe to make the solution uniform, 1 mL of citifluor was added, and the plates were observed under Fluorescence Microscopy (Nikon Eclipse 90i), (Minato, Tokyo, Japan)

### 2.9. Scanning Electron Microscope (SEM-EDS)

On the plates (TGO360 and K-562-cells/TGO360), surface metallization with gold was carried out (Denton Vacuum Model Desk IV equipment) at a pressure of 50 mTorr and a time of 60 s to generate a conductive surface, to later carry out scanning electron microscope (JEOL Model JSM 6490 LV), JEOL Ltd. (Japan Electron Optics Laboratory), (Akishima, Tokyo, Japan), inspection in backscattered electron mode to obtain a three-dimensional view of the topography of cells attached to the electrode surface.

### 2.10. Assembly of the Electrochemical Cell Biosensor

A Teflon electrochemical cell was used. This cell allows the contact area of the working electrode (WE) and the electrolytic solution to always be 0.41 cm^2^ and the volume to be a maximum of 1 mL. A reference electrode (Ag/AgCl, 013393 RE-1S, BAS Inc., Sumida-ku, Japan), TGO360 WE, and a 99.9% platinum counter electrode were installed at fixed distances to ensure consistent detection and allow rapid measurements with fewer cells.

### 2.11. Electrochemical Tests

The biosensor was assembled using three electrodes: a 3M Ag/AgCl reference electrode (RE), platinum counter electrode (CE), and TGO360 WE. Dilutions of concentrations of 1.0 × 10^6^ cells/mL, 5 × 10^5^ cells/mL, 1.0 × 10^5^ cells/mL, 2.5 × 10^4^ cells/mL, 6250 cells/mL and 1562 cells/mL were prepared in an Iscover medium using a micropipette. The desired concentrations were prepared, and the cells were placed in each concentration. The electric current response was characterized using a commercial Autolab B.V/Metrohm potentiostat by means of the differential pulse voltammetry (DPV)) technique, applying a pulse amplitude of 0.25 with a potential range (−0.20 to 0.20 V) at time intervals of 0.05 s.

## 3. Results

The sensitivity of the biosensor is dictated by the electrochemical response of the redox-active species during DPV, which reflects the electron transfer kinetics at the electrode interface. FTIR and SEM analyses confirmed the successful electrode functionalization and deposition of TiO_2_-m. To verify proper biofunctionalization, additional surface characterization steps were conducted to assess the adhesion and integrity of K-562 cells on the TGO360 electrode (K-562/TGO360). Leukemia cells attached to the TGO360 surface, displaying membrane elongation and pseudopod formation, indicative of active cell–surface engagement. These interactions facilitate stable and reproducible cell–electrode contacts, enabling reliable electrochemical signal transduction.

### 3.1. Characterization of the Electrode Materials

**Figure 1:**
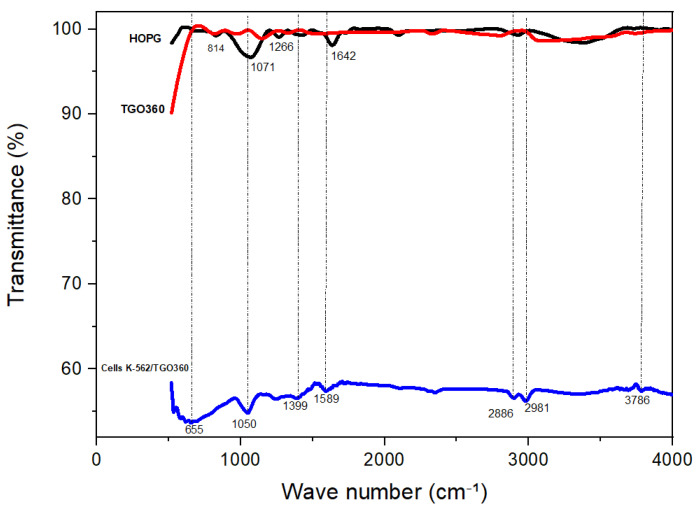
FTIR spectra of HOPG, TGO360, cells K-562/TGO360 in the 4000–400 cm^−1^ range.

The FTIR spectrum of HOPG (black line in Figure 1) shows a characteristic peak at 3362.28 cm^−1^ corresponding to O–H stretching, indicating the presence of hydroxyl groups; a peak at 2908.57 cm^−1^ attributed to C–H stretching vibrations, suggesting hydrocarbon groups on the surface; and a peak at 1693.98 cm^−1^ corresponding to C=C bond vibrations, characteristic of the graphitic structure with conjugated double bonds.

In contrast, the FTIR spectrum of the TGO360 plate (red line in Figure 1) shows a characteristic peak at 2796.95 cm^−1^ assigned to C–H stretching vibrations, indicating hydrocarbon groups in the material; a peak at 1144.83 cm^−1^ associated with C–O stretching; and a peak at 819.80 cm^−1^ corresponding to Ti–O–Ti.

Furthermore, the FTIR spectrum of the K-562/TGO360 electrode (blue line in Figure 1) shows a peak at 3786 cm^−1^ characteristic of the hydroxyl group, stretching bands between 2981 and 2886 cm^−1^ assigned to C–H stretching (possibly organic residues), a peak at 1589 cm^−1^ corresponding to C=C vibrations, and a peak at 1050 cm^−1^ associated with C–O stretching (from alcohols, ethers, or epoxides). Similarly, a peak at 655 cm^−1^ was observed, corresponding to Ti–O vibrations, which are indicative of the metallic bonds characteristic of Ti oxides [27].

### 3.2. Analysis of Cellular Behavior on the Electrode Material

**Figure 2:**
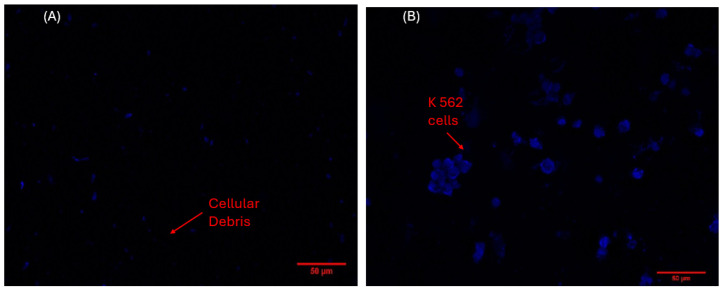
Fluorescence microscopy (**A**) HOPG plate with cell debris (**B**) TGO360 plate K562 cells adhered to the electrode.

Fluorescence microscopy was used to characterize the morphology and spatial distribution of K-562 cells, employing DAPI staining to visualize DNA nuclei through fluorescent emission. Figure 2a shows only cell debris, rather than intact cells, on the surface of the pyrolytic graphite (HOPG) electrode [25]. In contrast, Figure 2b clearly demonstrates that the K-562 cells adhered to the surface of the TGO360 electrode. The fundamental difference between HOPG and TGO360 is the presence of TiO_2_-m nanomaterials on the electrode surface. Additionally, rounded cells forming clusters were observed in regions where the nanomaterials were present.

**Figure 3:**
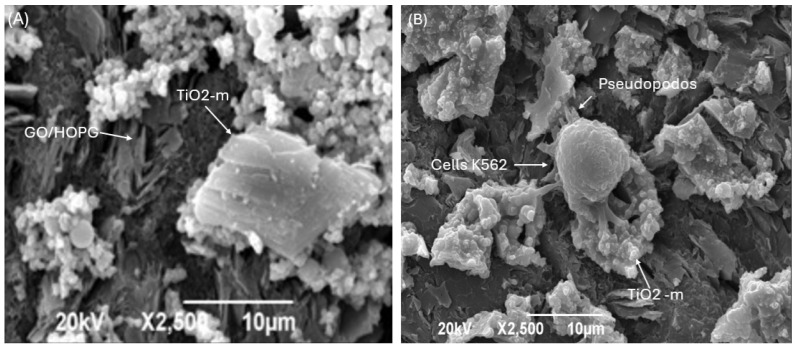
SEM at 2500× resolution (**A**) TGO360 without K-562 cells; (**B**) TGO360 with K-562 cells.

Figure 3 shows the SEM image of the TGO360 electrode. Figure 3a shows TGO360 before contact with K-562 cells; the electrode exhibited a non-uniform coating of TiO_2_-m (white accumulations). Figure 3b shows TGO360 that has been in contact with K-562 cells; the cells are attached to the TGO360 electrode, specifically on the TiO_2_-m; the cells exhibit elongations or stretches of the cytoplasmic membrane known as dendrites or pseudopods [26,31].

### 3.3. Electrochemical Detection

**Figure 4:**
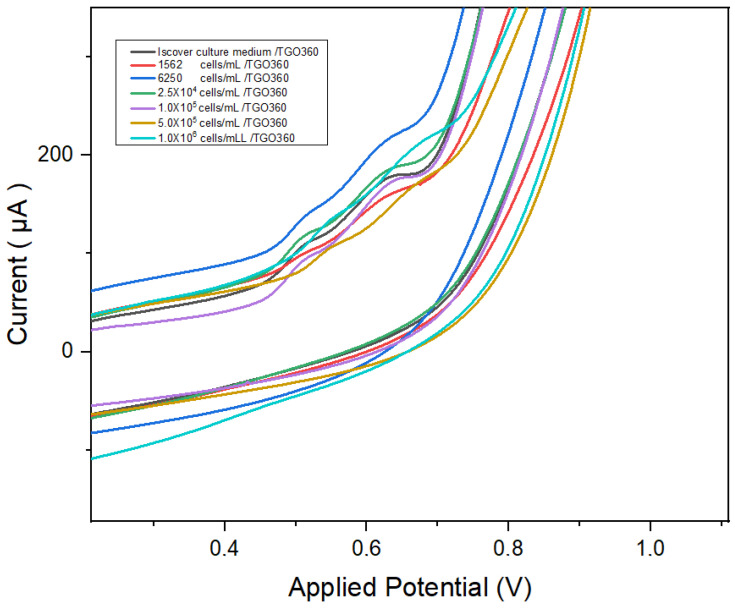
Cyclic voltammetry using Iscover medium and different concentrations [Cell/mL]/TGO360.

Figure 4 shows the current (I) curves as a function of the applied potential (V). It illustrates the signal variations in the cyclic voltammetry (CV) [32] response of the TGO360 electrode, with ranges of 0.9616–0.1893 at a scan rate of 0.01 V/s. The TGO360 plate was used as a control with Iscove’s culture medium. Electrochemical tests were performed at different concentrations of K- 562 cells/mL using Iscove’s medium as the electrolyte. Measurements were performed at different scan rates [33].

As shown in Figure 4, at a concentration of 6250 cells/mL, oxidation occurred at a current intensity of 2.12 × 10 A. At 2.5 × 10^4^ cells/mL, the current response was 1.84 × 10^−4^ A, whereas at 1,000,000 cells/mL, two oxidation peaks appeared (1.31 × 10^−4^ and 2.05 × 10^−4^ A). This indicates that the cells contributed to the conductivity and redox processes on the TGO360 electrode surface.

A review of the literature indicates that the DPV technique is one of the most sensitive and selective methods for cell detection, capable of detecting concentrations from 10 to 1.0 × 10^4^ cells/mL, with a detection limit as low as 10 cells/mL. It is also recognized as a highly sensitive technique for detecting low concentrations of biomolecules, such as 10 ng/mL PSA [34,35].

**Figure 5:**
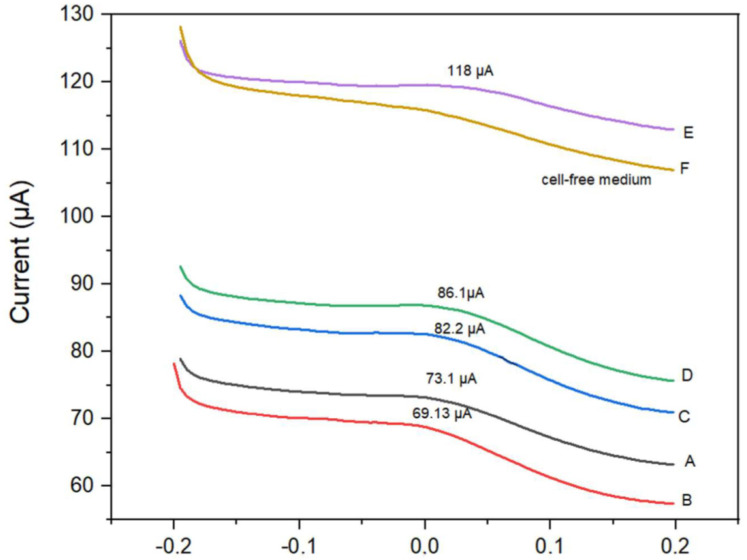
Current-potential curves obtained by differential pulse voltammetry to different concentrations of K-562/LCM cells: (A) 6250 cells/mL; (B) 2.5 × 10^4^ cells/mL; (C) 1.0 × 10^5^ cells/mL; (D) 5.0 × 10^5^ cells/mL; (E) 1.0 × 10^6^ cells/mL; (F) concentrations of 0 cells.

As seen in Figure 5 shows the response of only one of the three DPV replicates prepared for each cell/mL concentration. At 6250 cells/mL, the peak current was 73.1 µA, which was associated with oxidation–reduction between the cells and the TGO360 electrode. When the concentration increased to 2.5 × 10^4^ cells/mL, the signal decreased to 69.13 µA, indicating electron transfer at the surface of the modified electrode. At concentrations between 1.0 × 10 ^5^ and 5.0 × 10^5^ cells/mL, the signal stabilized between 82.2 and 86.1 µA, suggesting a surface saturation effect. Finally, at 1.0 × 10^6^ cells/mL, the signal reached 118 µA, indicating the formation of a conductive network that enhanced electron transfer. It is worth noting that the DPV curve of cell-free medium did not show a current peak. In the present investigation, DPV was the only electrochemical technique that allowed correlation between the concentration of K-562 cells and the current signal at the peak of each DPV. Similar use of DVP for detection of substances and genes has been reported [36,37].

**Figure 6:**
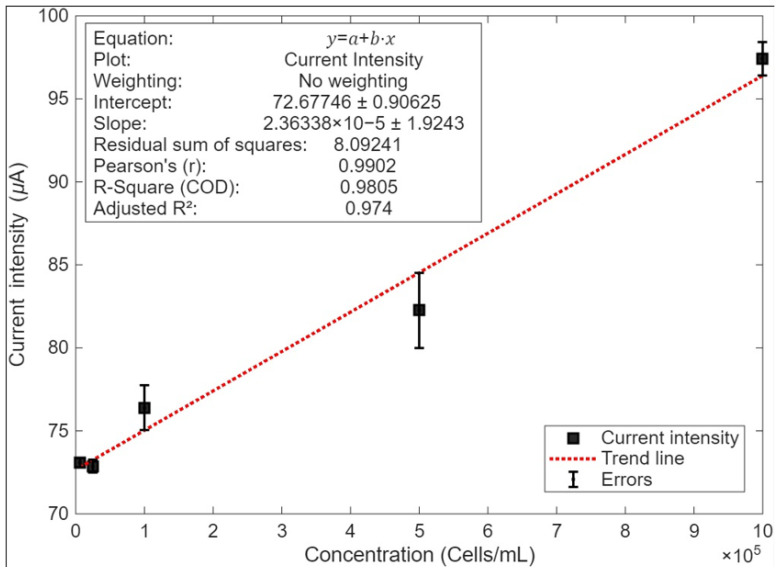
Calibration curve of the K-562 cells/TGO360 electrode.

The concentration range of 6250 to 1 × 10^6^ cells/mL showed reproducibility and sensitivity for cancer cell detection. Figure 6 highlights that the calibration was performed with the average values of the three replicates, obtaining an R^2^ of 98% and a calibration curve of y = 2 × 10^−5^ x + 72,677, as shown in Figure 6, with a detection time of 25 s.

The detection time, concentration range, and fabrication process of the biosensor demonstrated its ability to detect K-562 cells.

## 4. Discussion

K-562 cells are inherently non-adherent and typically remain suspended in the culture media. However, SEM–EDS analyses revealed that these cells acquired an adherent phenotype upon interaction with the TiO_2_-m nanomaterial, suggesting an interaction mechanism governed by the specific physicochemical properties of the nanostructured surface of the material. This behavior has been previously reported by Diosa et al., who demonstrated that non-adherent leukemia cell lines, such as Molt-4, which are representative of acute lymphoblastic leukemia, exhibit selective adhesion in the presence of the same nanomaterial. In contrast, healthy lymphocytes did not display similar adhesion, highlighting the intrinsic selectivity of TiO_2_-m for leukemic cell phenotypes [38]. Consequently, this selective interaction constitutes a significant advantage for electrochemical sensing applications, as it eliminates the need for additional biological recognition elements and suggests a preferential affinity between TiO_2_-m and cancer-altered cell membranes.

Multiple voltammetric techniques were used to evaluate the electrochemical response as a function of the cell concentration. Although CV produced detectable signals in the presence of cells, it did not show a proportional or reproducible trend that reliably correlated with variations in the cell concentration. In contrast, DPV exhibited a clear, sequential, and concentration-responsive current profile, establishing it as the most suitable transduction technique for this sensor. The DPV-based biosensor demonstrated a wide linear detection range (10 to 1 × 10^4^ cells/mL), sensitivity, and reproducibility, supported by signal dispersion and a linear correlation in the calibration curve [39]. The DPV-based biosensor demonstrated a wide linear detection range, an electrochemical response dependent on cell concentration, and adequate reproducibility for a whole-cell-based system, supported by a consistent linear correlation in the calibration curve. This linearity falls within the range reported for other electrochemical biosensors, including both label-based detection strategies and label-free approaches, as well as systems incorporating complex recognition and amplification architectures [35,40,41,42,43,44,45].

Additionally, morphological analysis showed that adherent cells exhibited diameters ranging from 8.5 to 9.9 µm, and that the TiO_2_-m coating promoted stable cell–surface interactions. This was likely due to the increased effective surface area and surface chemistry of the nanostructures [45].

The sensor achieved a response time of 25 s, which represents a substantial advantage over conventional diagnostic methods such as ELISA and PCR. These technologies typically require long processing times, complex preparation steps or molecular labeling [37]. Although other advanced biosensors, such as antibody-functionalized gold electrodes, have similar detection limits (~10 cells/mL), they often require complex biorecognition layers that increase the cost and limit scalability. In contrast, the TGO360 platform achieved competitive sensitivity without requiring specific biorecognition elements, representing an advancement in label-free cell detection methods [46,47].

Electrochemical measurements further confirmed that the sensor signal was exclusively associated with the presence of cells, as no peaks were observed in cell-free controls. Moreover, the systematic peak potential shift and current increase at higher cell concentrations indicated that cell adhesion modulated the electron transfer kinetics at the TGO360 interface. According to the literature, living cells possess endogenous redox centers linked to membrane proteins, enzymes, and intracellular metabolic complexes that can participate in charge transfer processes when interfaced with electroactive surfaces. It is worth noting that this type of direct electrochemical transduction based on the intrinsic electrochemical [48] activity of cells has been reported in a limited number of studies In this context, the cell–nanomaterial interaction favors a less explored interfacial mechanism that promotes electron transfer. These phenomena, together with the physiological changes associated with cellular proliferation, appear to contribute to the electrochemical responses observed in this system [35,36]. Thus, this platform not only enables cell detection but also supports the direct transduction of biological redox activity without the need for external redox probes or labels.

The observed electrochemical signal was likely the result of several synergistic factors. First, the adhesion of K-562 cells, facilitated by the specific physicochemical properties of the TiO_2_-m surface and evidenced by pseudopod formation, brings the cell membrane close to the electrode. This intimate contact enables electron transfer from endogenous redox-active biomolecules within or on the cell membrane (e.g., membrane-bound enzymes, metabolic intermediates, or components of the electron transport chain) to the TGO360 interface. The observed increase in current with cell concentration (Figure 6), up to saturation, suggests that a greater number of adhered cells contribute more redox-active sites to the electrode surface, thereby enhancing the overall electrochemical signal. The specific nature of these redox species and their interactions with the TiO_2_-m surface warrant further investigation but likely involve direct charge transfer or mediated processes facilitated by the semiconducting properties of the nanomaterial.

Taken together, these findings establish the TGO360 biosensor as a promising platform for the development of rapid, portable, and highly sensitive diagnostic devices. Furthermore, it offers potential for differential detection across malignant cell lines, long-term stability studies and eventual clinical validation.

## 5. Conclusions

The electrochemical biosensor based on TGO360 proved to be a sensitive, reproducible, and label-free platform for detecting K-562 leukemic cells, enabling the direct electrochemical transduction of cellular redox processes without the need for external mediators or biorecognition layers.

Typically non-adherent, K-562 cells exhibited selective adhesion to the TiO_2_-m interface, as confirmed by SEM-EDS and fluorescence microscopy, indicating that the nanostructured surface promotes specific cell–material interactions, likely governed by the physicochemical affinity between cancer-altered membranes and nanoscale coating. This demonstrates the inherent selectivity of the method, without the need for additional biological functionalization.

CV revealed the baseline electrochemical activity, whereas DPV provided a significantly more reliable and concentration-dependent response, exhibiting a linear correlation (R^2^ ≈ 0.98) across a dynamic detection range, confirming it as the most suitable transduction method for this biosensing architecture.

The device achieved a response time of 25 s, outperforming conventional diagnostic techniques such as ELISA and PCR and delivering sensitivity comparable to more complex antibody-modified platforms, but with the critical advantages of label-free detection, simpler fabrication, low cost, and scalability.

The observed increase in the peak current and potential shift was attributed to endogenous cellular redox activity (membrane proteins, enzymes, and metabolic electron transfer systems), confirming that the biosensor directly interrogates biological electrochemical signals rather than nonspecific bulk effects.

Overall, this study demonstrates the TiO_2_-m/GO/HOPG system as a candidate for rapid, label-free electrochemical detection of leukemic cells, integrated into portable point-of-care diagnostic devices.

## Data Availability

The original contributions presented in this study are included in the article. Further inquiries can be directed to the corresponding author.
